# Case Report: Pulmonary Conidiobolomycosis in a Vietnamese Pot-Bellied Pig

**DOI:** 10.3389/fvets.2021.799641

**Published:** 2021-12-20

**Authors:** Brittany L. Rasche, Samuel M. Tucker, Keith Linder, Tara M. Harrison, Tatiane Terumi Negrão Watanabe

**Affiliations:** ^1^Department of Population Health and Pathobiology, College of Veterinary Medicine, North Carolina State University, Raleigh, NC, United States; ^2^Department of Clinical Sciences, College of Veterinary Medicine, North Carolina State University, Raleigh, NC, United States

**Keywords:** *Conidiobolus* spp., entomophthoromycosis, pneumonia, domestic animal, pig

## Abstract

An adult castrated male Vietnamese pot-bellied pig had a 1-week history of acute dyspnea and lethargy. Minimal diagnostic testing was authorized by the owner, resulting in treatment with a third-generation cephalosporin and a non-steroidal anti-inflammatory drug. Partial improvement was observed after a week; however, the pig died 2 weeks after the initial onset of clinical signs. Macroscopically, ~90% of the left lung was effaced by large masses with a caseonecrotic center. Histologic examination revealed eosinophilic granulomas with myriad, intralesional, negatively staining hyphae highlighted by “sleeves” of hypereosinophilic material (Splendore-Hoeppli material). Infection with an oomycete or “zygomycete” (i.e., organisms of the order Entomophthorales or Mucorales) was initially considered. Pan-fungal PCR and sequencing performed on formalin-fixed, paraffin-embedded lung tissue identified *Conidiobolus* spp., consistent with a diagnosis of primary pulmonary conidiobolomycosis. There are only a few reports of infections with *Conidiobolus* spp. (and other members of the order Entomophthorales) in swine. Unlike humans and other animal species, conidiobolomycosis in pigs presents more commonly as a primary pulmonary disease rather than rhinofacial or nasopharyngeal disease.

## Introduction

*Conidiobolus* spp. are members of the order Entomophthorales, subphylum Entomophthoromycotina, and the phylum Glomeromycota (1). Previously, *Conidiobolus* spp. were classified in the phylum Zygomycota and were referred to as “zygomycetes” along with a variety of other fungal species, including those in the order Mucorales. However, studies revealed the phylum Zygomycota to be polyphyletic, so the phylum was renamed Glomeromycota and split into four subphyla, including Entomophthoromycotina and Mucoromycotina (1, 2). Thus, the term “zygomycosis” has been replaced by “entomophthoromycosis” and “mucormycosis” (1, 2). Being saprobes, these fungi are often found in decaying plant matter and soil, especially in tropical and subtropical climates (2). They can also exist as facultative or obligate insect pathogens (2). Among the genus *Conidiobolus, C. coronatus, C. incongruus*, and *C. lamprauges* present as specific species of pathological importance in humans and animals, including dogs, horses, sheep, goats, deer, llamas, pigs, and non-human primates (3–7). *Conidiobolus* spp. infections seem to occur more frequently in livestock and humans than in domestic canines and felines (8). The primary modes of transmission include inhalation, ingestion, or dermal exposure to spores *via* minor trauma or insect bites (2, 8).

## Case Description

An ~3-year-old (exact age unknown), 43.6-kg castrated male Vietnamese pot-bellied pig was referred to the North Carolina State University Exotic Animal Medicine Service with a 1-week history of lethargy and acute onset of dyspnea in the mid to late summer. The pig was housed with two other apparently unaffected pigs in an air-conditioned outdoor shed with backyard access. No history of recent travel was reported. On physical examination, moderate tachypnea with increased respiratory effort and mild bilateral serous to mucoid nasal discharge were noted. Subtle crackles were present over the left lung fields. Due to financial constraints, further diagnostic testing was not performed. One intramuscular injection of a third-generation cephalosporin (ceftiofur–5 mg/kg IM) was administered in the hospital and the animal was discharged with a prescription of a non-steroidal anti-inflammatory drug (carprofen–2.2 mg/kg PO q12h) for 7 days. The patient seemed to clinically improve over the first week; however, during the administration of the second injection of ceftiofur 1 week after the initial presentation, the pig went into acute respiratory distress and died shortly afterward.

**Figure 1:**
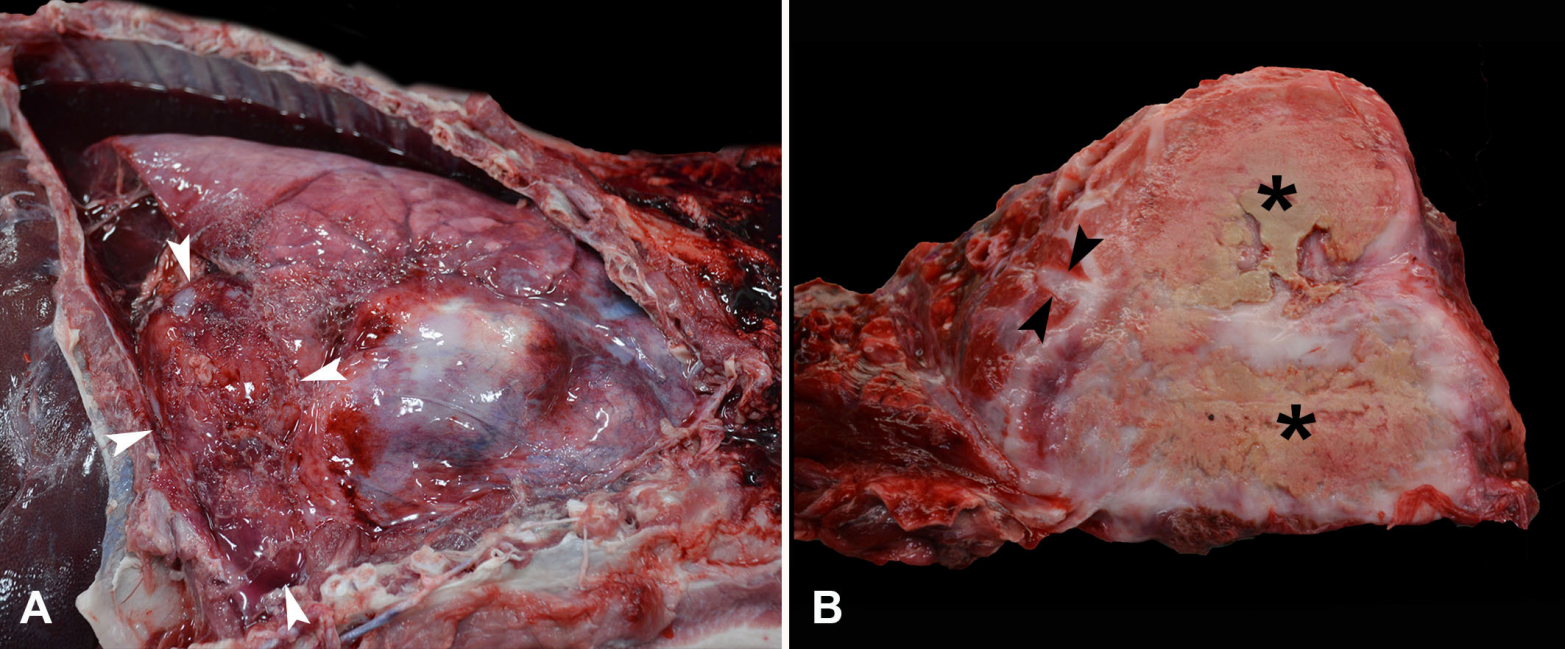
Photographs from gross post-mortem examination of an adult castrated male Vietnamese pot-bellied pig with acute dyspnea and lethargy. **(A)** The pleural cavity contains a moderate amount of dark red serosanguinous effusion with abundant fibrin along the pleura of the left lung lobes, ventral right lung lobes, and adjacent pericardial sac. The left caudal lung lobe (denoted by white arrowheads) is markedly expanded and displaced to the right of the midline. **(B)** On cut surface, the left lung lobe is markedly expanded by multifocal granulomas with dry yellow to tan caseonecrotic centers (*) surrounded by firm, smooth, pale tan, fibrous tissue that blends into adjacent pulmonary parenchyma. The interlobular septa of the adjacent lung are moderately expanded with edema (arrowheads).

At necropsy, the pleural cavity contained ~300 ml of dark red, watery fluid. Approximately 90% of the left cranial and caudal lung lobes were replaced by two, large, firm, tan masses measuring 12 × 11 × 6 cm and 14 × 13 × 12 cm (Figure 1A). The pulmonary masses occupied ~70% of the thoracic cavity and displaced the mediastinum to the right of the midline, resulting in compressive atelectasis of the right lung. The cranial pulmonary mass was firmly adherent to the thoracic wall at the level of the left second and third ribs. Multiple fibrinous adhesions were present from both pulmonary masses and the parietal pericardium to the left parietal pleura. On cut surface, the pulmonary masses had a dry, yellow to tan, malodorous caseonecrotic center surrounded by abundant firm, smooth, pale tan, fibrous tissue that blended into the adjacent pulmonary parenchyma (Figure 1B). The adjacent interlobular septa were moderately expanded by edema (Figure 1B). The sternal, gastric, and peripancreatic lymph nodes were diffusely moderately enlarged with the maintenance of typical architecture. The peritoneal cavity contained 130 ml of pale red-orange, watery fluid with scant fibrin. Multifocal fibrous adhesions were present between the liver and the diaphragm.

Touch impression cytology of the pulmonary masses revealed a mixture of foamy macrophages, lymphocytes, plasma cells, eosinophils, and few multinucleated giant cells, suggestive of granulomatous inflammation. Given the gross appearance as caseous pulmonary granulomas, pulmonary mycobacteriosis was initially considered as an important differential with zoonotic potential. However, granulomatous lymphadenitis, a common lesion of porcine mycobacteriosis, was not grossly observed.

Tissue samples of all parenchymal organs were fixed in 10% neutral buffered formalin, processed routinely, and stained with hematoxylin and eosin. Fresh samples of the pulmonary masses were submitted for aerobic bacterial culture, *Mycobacterium-*specific culture, PCR for *Mycobacterium* spp., and fungal culture.

**Figure 2:**
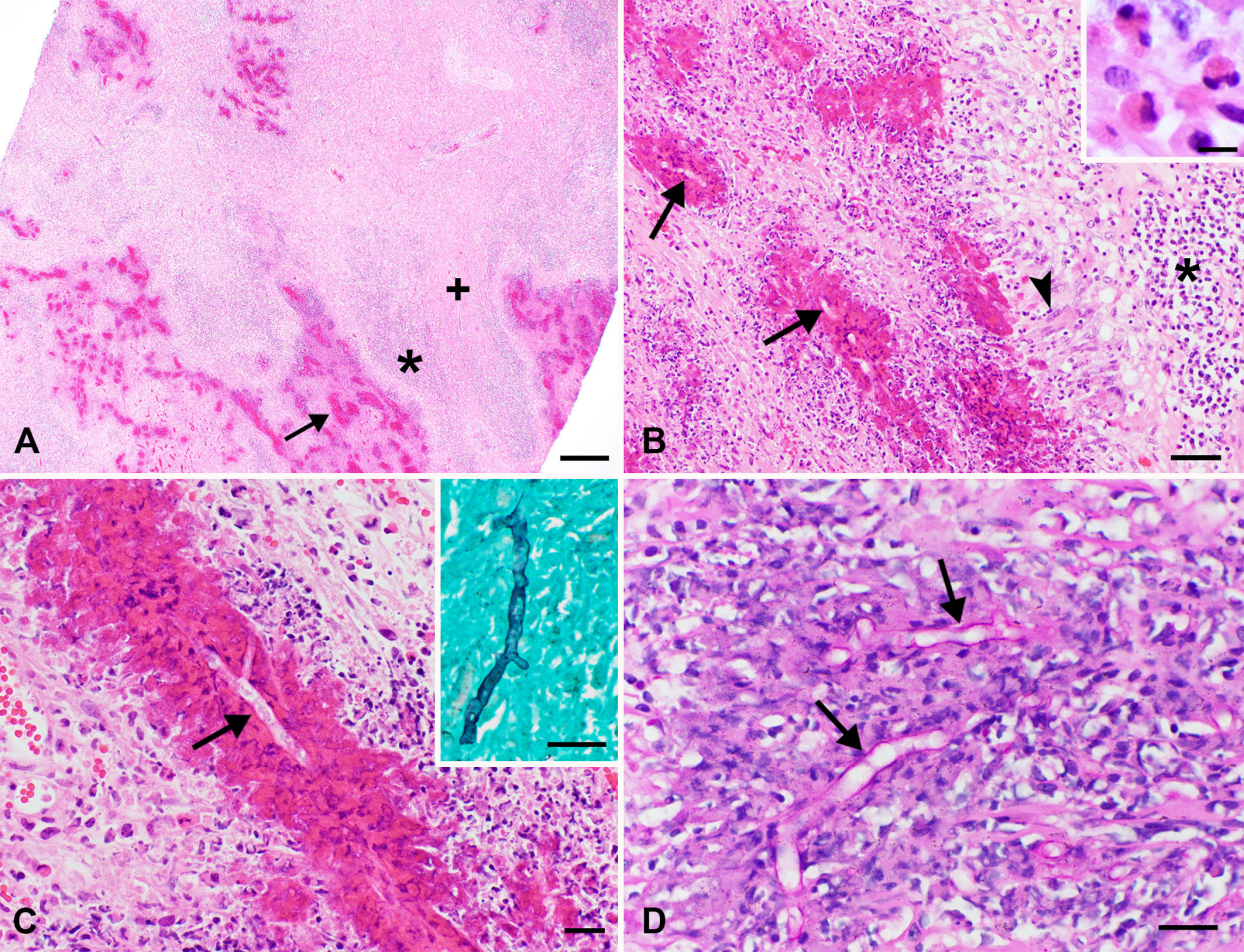
Photomicrographs of sections of lung obtained from the pig in Figure 1. **(A,B)** The pulmonary parenchyma is largely effaced by multifocal to coalescing granulomas centered around non-staining hyphae highlighted by bright eosinophilic “sleeves” of Splendore-Hoeppli material (arrows) with degranulating and intact eosinophils (highlighted in the inset). Surrounding this is a layer of multinucleated giant cells and epithelioid macrophages (arrowhead), which are bordered by a thick band of lymphocytes and plasma cells (*). Coalescing between these granulomas is abundant dense fibrous stroma (+). H&E stain; bar = 500 μm **(A)**, 50 μm **(B)**, 10 μm **(B inset)**. **(C)** Higher magnification view showing 7–14 μm diameter, non-staining hyphae (arrow) with occasional non-dichotomous branching highlighted by brightly eosinophilic sleeves. H&E stain; bar = 20 μm. The inset illustrates positive staining of hyphae with GMS. Bar = 20 μm. **(D)** Hyphae (arrows) also stain weakly positive with PAS, which illustrates the infrequent septation and non-parallel walls of these hyphae. Bar = 20 μm.

On histologic examination, the left pulmonary masses consisted of multifocal to coalescing eosinophilic granulomas that almost completely effaced and replaced the left pulmonary parenchyma. The eosinophilic granulomas were centered on myriad, 7–14 μm diameter, non-staining fungal hyphae delineated by “sleeves” of brightly eosinophilic material (Splendore-Hoeppli material) and surrounded by several degranulating and viable eosinophils, followed by a layer of epithelioid macrophages and multinucleated giant cells with up to 9 nuclei. Lymphocytes and plasma cells were peripherally placed and merged with a dense fibrous capsule (Figures 2A,B). The hyphae were positively stained with Gomori methenamine-silver (GMS) stain and weakly stained with Periodic acid-Schiff (PAS) stain. These stains highlighted additional features of the fungi, including non-parallel walls, infrequent septation, and occasional non-dichotomous branching (Figures 2C,D). Ziehl-Neelsen stain was negative for acid-fast bacteria (such as *Mycobacterium* spp.) in the lungs. In the remaining less-affected pulmonary parenchyma, the interlobular septa, alveoli, and bronchiolar lumens contained abundant fibrin exudates and edema intermixed with variable numbers of eosinophils, lymphocytes, plasma cells, and macrophages.

Histologic examination of the enlarged lymph nodes revealed increased numbers of prominent secondary lymphoid follicles with a mild increase in supporting fibrous stroma (reactive node with lymphoid hyperplasia). In sections of the liver, the portal tracts and interlobular septa were multifocally infiltrated by low to moderate numbers of eosinophils, lymphocytes, and plasma cells, consistent with non-specific reactive hepatitis. Mild centrilobular to mid-zonal hepatic cord atrophy was present with a small amount of fibrin attached to multifocal areas of the hepatic capsule. Multifocally infiltrating the interstitium of the renal cortex, especially around blood vessels, were low numbers of lymphocytes and plasma cells (mild non-specific interstitial nephritis).

Despite the identification of hyphae histologically, the fungal culture of the lung yielded no growth. To further classify this microorganism, formalin-fixed, paraffin-embedded (FFPE) lung tissue was submitted to the Texas A&M Veterinary Medical Diagnostic Laboratory for pan-fungal PCR and sequencing. PCR targeting the internal transcribed spacer (ITS) region and the large subunit (LSU) region yielded 1 band of DNA from each region at ~450 and 1,000 bp *via* gel electrophoresis. DNA was purified from the gel, sequenced, and analyzed with the NCBI BLAST database. The LSU sequence matched *Conidiobolus* spp. with 100% identity. The ITS sequence was of poor quality and failed to match any specific fungi with more than 97% identity. Thus, PCR and sequencing in combination with histology confirmed a diagnosis of pulmonary conidiobolomycosis in this pig.

The aerobic bacterial culture of the lung revealed growth of *Lactobacillus salivarius* in thioglycolate broth only, which was considered a contaminant. *Mycobacterium* spp. was not detected on culture or PCR from fresh pulmonary tissue.

## Discussion

As previously noted in the case description, one important potentially zoonotic differential for the gross appearance of large pulmonary masses with central necrosis was pulmonary mycobacteriosis. However, granulomatous lymphadenitis, a primary lesion of porcine mycobacteriosis, was not present in this case (9). Additionally, *Mycobacterium* spp. was not detected on culture, PCR, or acid-fast stain of the lung tissue. Other reported causes of granulomatous pneumonia in swine, including certain bacteria (i.e., *Actinobacillus porcitonsillarum*), viruses (i.e., porcine circovirus 2), parasites (i.e., *Metastrongylus* spp.), and fungi (i.e., *Pneumocystis carinii, Conidiobolus* spp., *Mucor* spp.) may also be considered as differential diagnoses (10, 11). However, some of these differentials typically present as a more generalized interstitial pneumonia (porcine circovirus 2 and pneumocystosis) or small (<5 mm in diameter) pulmonary nodules (*Metastrongylus* spp.) in contrast to the large discrete pulmonary masses observed in this case (10). In the single case report of *Actinobacillus porcitonsillarum* infection in a pig, there was granulomatous inflammation observed in pulmonary hilar and mediastinal lymph nodes as well as in the lungs. Interestingly, this type of cellular infiltrate was centered on irregular aggregates of brightly eosinophilic material (Splendore-Hoeppli material); however, it was occasionally associated with bacterial colonies rather than hyphae (11). Granulomatous pneumonia caused by oomycetes may also be considered as a differential diagnosis given the histologic identification of non-staining hyphae. However, there are only rare reports of a primary pulmonary presentation of pythiosis (one jaguar and two South American coatis) (12, 13) and of dissemination of lagenidiosis to the lung (two dogs) (14) in veterinary literature. Another differential for large pulmonary masses in any species is primary pulmonary neoplasia. However, pulmonary neoplasia is exceedingly rare in pigs with few reports of metastatic lymphoma involving the lungs and single reports of bronchogenic carcinoma, chondrosarcoma, fibrosarcoma, and malignant peripheral nerve sheath tumor (15, 16).

Mycotic pneumonia is infrequently encountered in pigs with *Pneumocystis carinii*, a ubiquitous and opportunistic pathogen, most frequently implicated. In contrast to the lesions observed in this case, pneumocystosis typically presents as a multifocal to diffuse interstitial pneumonia with round to crescent-shaped intra- and extracellular fungal cysts observed on histology (10). More recently, there have also been reports of pulmonary entomophthoromycosis or mucormycosis, which were previously known as zygomycosis, in pigs. Four cases of pulmonary entomophthoromycosis and mucormycosis in Vietnamese potbellied pigs have been described and attributed to *C. incongruus, Mucor circinelloides*, and *Peniophora* spp. (3, 17). All of these pigs were presented with respiratory signs and pleural effusion and had one or more large pulmonary granulomas identified at necropsy. Microscopically, these granulomas contained fungal hyphae surrounded by brightly eosinophilic material (Splendore-Hoeppli material and/or degranulating eosinophils), similar to the case presented here (3, 17). In addition to pneumonia, entomophthoromycosis or mucormycosis in swine has also been implicated in submandibular abscesses, mesenteric lymphadenopathy, hepatic granulomas, and gastritis (18, 19).

In other species (humans, horses, sheep, goats, and dogs), conidiobolomycosis typically presents as rhinofacial or nasopharyngeal lesions (4, 8, 20, 21). In sheep, as shown in endemically infected populations in Brazil, conidiobolomycosis often results in non-specific clinical signs of malaise, anorexia, weight loss, and serous/mucoid to hemorrhagic nasal discharge (7, 20, 22). Disseminated/systemic conidiobolomycosis is less commonly reported but has been described in humans, sheep, dogs, deer, and pigs (5, 6, 18, 21, 22). In one of these reported cases of disseminated conidiobolomycosis from a North American white-tailed deer, there was a large intrathoracic mass associated with the accessory lung lobe consisting of eosinophilic granulomatous inflammation, potentially suggestive of a primary pulmonary lesion (6). Reports of primary pulmonary conidiobolomycosis are rare, including two dogs and the two aforementioned pigs (3, 5, 17, 23). The case reported herein is thought to represent a primary pulmonary conidiobolomycosis since dissemination of conidiobolomycosis to other organs was not observed grossly or histologically. Examination of the nasal cavity was not performed at necropsy due to zoonotic concerns and additional nasal cavity involvement could not be completely ruled out.

Due to non-specific clinical presentation, a variety of diagnostic tests are often needed to reach an antemortem diagnosis of conidiobolomycosis. Diagnostic imaging [radiographs, rhinoscopy, bronchoscopy, ultrasonography, or computed tomography (CT)] may be helpful in identifying nodular lesions in the respiratory tract and/or pleural effusion (3, 17). Diagnostic samples may be obtained by biopsy, tracheal wash, or bronchoalveolar lavage for fungal culture, cytology, and histopathology (5, 23, 24). Fungal cultures can have slow turnaround times and have repeatedly yielded false negatives in cases of conidiobolomycosis (17, 21, 25). Failure to culture *Conidiobolus* spp. and related organisms may be due to improper incubation temperature, as one study showed enhanced growth of these fungi at an incubation temperature of 37°C vs. customary room temperature incubation at 25°C (25). Impaired growth of these fungi at room temperature may explain the negative fungal culture in this case. Recommendations for fungal culture media for *Conidiobolus* spp. are Sabouraud dextrose agar (SDA), potato dextrose agar (PDA), or cornmeal agar, which will typically display growth after 5 days at 37°C (7, 21).

In cases of conidiobolomycosis, diagnostic cytology (from a touch impression of a biopsy sample or from fluid from a tracheal wash, bronchoalveolar lavage, or thoracocentesis) may show eosinophilic, suppurative, and/or granulomatous inflammation with or without fungal hyphae (3, 5, 8, 23). Typical histologic lesions of conidiobolomycosis in all veterinary species consist of pyogranulomatous to granulomatous inflammation, often with a prominent eosinophilic inflammatory infiltrate (3, 6, 8, 10, 17, 20). This inflammation is centered around non-staining to slightly basophilic fungal hyphae that are surrounded by thick “sleeves” of amorphous to granular eosinophilic material, which represents Splendore-Hoeppli material (antigen-antibody complexes) and potentially eosinophil major basic protein from degranulating eosinophils (3, 6, 10, 20, 22, 26, 27). The Splendore-Hoeppli reaction is a characteristic feature of *Conidiobolus* spp. infection but can also be observed with infection by other members of the orders Entomophthorales and Mucorales as well as other types of infectious agents (including certain bacteria and parasites) (3). GMS stain highlights the hyphae and helps to reveal occasional septation and infrequent non-dichotomous branching; whereas, the hyphae often do not stain or stain only weakly with PAS stain (8, 20).

Similar histologic findings can also be observed in oomycosis, such as pythiosis or lagenidiosis; however, oomycosis most commonly presents as a cutaneous or gastrointestinal disease with rare reports involving the lungs (12–14). One potential differentiating feature is that hyphae of *Pythium insidiosum* may be slightly smaller (2–7 μm) than those of *Conidiobolus* spp. (5–20 μm) (8, 10). However, *Lagenidium* spp. has 7–25 μm wide hyphae that more closely overlap in size with *Conidiobolus* spp., but lagenidiosis has only been reported in humans, dogs, and cats to our knowledge (8, 28). Given the overlap in histologic features between entomophthoromycosis, mucormycosis, and oomycosis, fungal culture and/or PCR with DNA sequencing are required for definitive diagnosis (3, 10, 17). Panfungal PCR and DNA sequencing can be performed on fresh or formalin-fixed paraffin-embedded tissue, as in this case.

Due to the low number of reported cases of conidiobolomycosis, there is limited evidence for effective treatments (5, 20). Complete and aggressive surgical excision of granulomas with accompanying long-term antifungal therapy (such as itraconazole) is considered the gold standard for treatment in many species, including humans and dogs (5, 21). If surgical excision is not feasible, the use of one or more antifungal medications has limited efficacy but did prove successful in one reported case of canine conidiobolomycosis (23).

Conidiobolomycosis in humans typically presents as chronic sinusitis with swelling and ulceration of the facial subcutaneous tissue and lining of the paranasal sinuses (2). Without treatment, generalized facial swelling and disfigurement with nasal congestion, sinus pain, and epistaxis may occur (2). Cases are most commonly reported in immunocompetent adults that live in tropical and subtropical locations, such as Africa, Southeast Asia, and Central and South America (2, 24, 26). To our knowledge, no cases of direct transmission between animals and humans have been reported. Currently, no drugs have proven to be effective in treating human cases but prolonged antifungal therapy and surgical debridement have been used with variable success (2, 21, 26).

## Conclusion

While usually encountered in more tropical locales, conidiobolomycosis can also occur in more temperate regions during the summer as shown in this particular case. Furthermore, while the majority of cases are localized to the rhinofacial or nasopharyngeal region, this case provides additional evidence of rare primary pulmonary presentation of conidiobolomycosis that can occur in domestic pigs. As more cases of pulmonary conidiobolomycosis are identified, the diagnostic approach and treatment may be further refined.

## Data Availability Statement

The original contributions presented in the study are included in the article/supplementary material, further inquiries can be directed to the corresponding author.

## Ethics Statement

The owners gave the permission for the post-mortem evaluation, even though ethical review and approval was not required for this case. The pig was submitted for routine diagnostic post-mortem examination to the Department of Pathobiology and as a result not subject to animal ethics guidelines.

## Author Contributions

TH followed the clinical case. BR and KL performed the post-mortem examination, sample collection, and final post-mortem report. BR and ST prepared the manuscript and literature review. TN contributed to the design, supervised the study, and critically revised and edited the manuscript. TH, BR, KL, ST, and TN reviewed the final submission. All authors read and approved the final manuscript.

## Conflict of Interest

The authors declare that the research was conducted in the absence of any commercial or financial relationships that could be construed as a potential conflict of interest.

## Publisher's Note

All claims expressed in this article are solely those of the authors and do not necessarily represent those of their affiliated organizations, or those of the publisher, the editors and the reviewers. Any product that may be evaluated in this article, or claim that may be made by its manufacturer, is not guaranteed or endorsed by the publisher.
